# Defect structure in δ-Bi_5_PbY_2_O_11.5_

**DOI:** 10.1039/c9ra01233h

**Published:** 2019-03-26

**Authors:** Anna Borowska-Centkowska, Xi Liu, Marcin Krynski, Marzena Leszczynska, Wojciech Wrobel, Marcin Malys, Stephen Hull, Stefan T. Norberg, Franciszek Krok, Isaac Abrahams

**Affiliations:** Faculty of Physics, Warsaw University of Technology ul. Koszykowa 75 00-662 Warszawa Poland centkowska@if.pw.edu.pl; Materials Research Institute, School of Biological and Chemical Sciences, Queen Mary University of London Mile End Road London E1 4NS UK i.abrahams@qmul.ac.uk; ISIS Facility, Rutherford Appleton Laboratory Chilton, Didcot Oxon OX11 0QX UK; Chalmers University of Technology, Department of Chemistry and Chemical Engineering SE-41296 Gothenburg Sweden

## Abstract

A detailed study of the defect structure in a di-substituted δ-Bi_2_O_3_ type phase, δ-Bi_5_PbY_2_O_11.5_, is presented. Using a combination of conventional Rietveld analysis of neutron diffraction data, reverse Monte Carlo (RMC) analysis of total neutron scattering data and *ab initio* molecular dynamics (MD) simulations, both average and local structures have been characterized. δ-Bi_5_PbY_2_O_11.5_ represents a model system for the highly conducting δ-Bi_2_O_3_ type phases, in which there is a higher nominal vacancy concentration than in the unsubstituted parent compound. Uniquely, the methodology developed in this study has afforded the opportunity to study both oxide-ion vacancy ordering as well as specific cation–cation interactions. Oxide-ion vacancies in this system have been found to show a preference for association with Pb^2+^ cations, with some evidence for clustering of these cations. The system shows a non-random distribution of vacancy pair alignments, with a preference for 〈100〉 ordering, the extent of which shows thermal variation. MD simulations indicate a predominance of oxide-ion jumps in the 〈100〉 direction.

## Abstract 2

Total neutron scattering analysis and *ab initio* MD simulations reveal details of oxide ion vacancy ordering and migration pathways.

## Graphical abstract

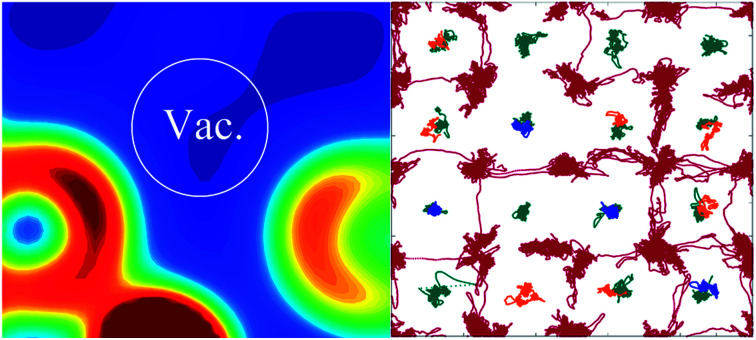

## 1. Introduction

Bismuth oxide and bismuth oxide based compounds are known for their catalytic,^1^ optical^2–4^ and oxide ion conducting^5–10^ properties. The high temperature δ-polymorph of bismuth sesquioxide (Bi_2_O_3_) is best known for its high oxide-ion conductivity at elevated temperatures. The structure of δ-Bi_2_O_3_ is that of a defect fluorite and can be described as a cubic close packed (ccp) lattice of Bi^3+^ ions, with O^2−^ ions disordered over ¾ of the tetrahedral sites. The exceptional oxide-ion conductivity of this phase is attributable to the high intrinsic oxide-ion vacancy concentration of 0.5 vacancies per metal atom with respect to the ideal fluorite structure, the high polarizability of the bismuth framework and the presence of three dimensional conduction pathways. Unfortunately, this phase is unstable below *ca.* 730 °C and readily transforms to other poorly conducting polymorphs on cooling.^11^ Preservation of the high-temperature phase to lower temperatures is most commonly achieved by partial substitution of bismuth by other cations and this is reviewed extensively elsewhere.^5–10^

We have recently investigated the ternary system Bi_2_O_3_–PbO–Y_2_O_3_, where Bi^3+^ is substituted for a combination of subvalent and isovalent cations (Pb^2+^ and Y^3+^), leading to δ-Bi_2_O_3_ type structures, with nominal vacancy concentrations higher than in the stoichiometric parent compound.^12^ Yttrium substitution for bismuth in Bi_2_O_3_ is well known to yield highly conducting cubic δ-type phases over a relatively large composition range^13–18^ and much of this work is reviewed by Sammes *et al.*^8^ In contrast, despite the close similarity of the Bi^3+^ and Pb^2+^ cations, in terms of their stereochemistries (dominated by the 6s^2^ lone pair) and polarizabilities, lead substitution for bismuth does not lead to the appearance of the δ-type phase at low temperatures.^19^ However, in ternary systems such as Bi_2_O_3_–MO_*x*_–PbO (M = Ca, Y, Er and La), preservation of the δ-type phase to room temperature is observed.^20–24^

In a previous study we examined compositions in the Bi_2_O_3_–PbO–Y_2_O_3_ system of general formula Bi_2.5+*x*_Pb_0.5_YO_5.75+3*x*/2_ (*x* = 0, 1 and 2), which showed the δ-phase structure.^12^ These phases exhibited very high conductivities (*ca.* 0.4 S cm^−1^ at 800 °C), with ionic transference numbers close to unity. In the present study, we examine the defect structure of the most lead-rich member of this series, Bi_5_PbY_2_O_11.5_, using neutron diffraction, analysed using both conventional Rietveld analysis and reverse Monte Carlo (RMC) modelling of total neutron scattering data. These results are compared to those from density functional theory (DFT) simulations to yield a comprehensive picture of the defect structure and oxide-ion motion in a model system for this important class of compounds.

## 2. Experimental

### 2.1 Sample preparation

The title compound was prepared using stoichiometric amounts of Bi_2_O_3_ (Sigma Aldrich, 99.9%), PbO (Sigma Aldrich, 99.99%) and Y_2_O_3_ (Sigma Aldrich, 99.99%). The starting mixture was ground in ethanol in an agate mortar and the dried mixture heated at 650 °C for 20 h, then cooled and reground. The sample was subsequently reheated to 800 °C for 24 h and quenched in air to room temperature.

### 2.2 Diffraction

Neutron powder diffraction data were collected on the Polaris diffractometer at the ISIS Facility, Rutherford Appleton Laboratory, on back-scattering (130–160°), 90° (85–95°), low-angle (28–42°) and very low angle (13–15°) detectors over the respective time of flight ranges 1.0 to 20 ms, 0.8 to 19.2 ms, 0.5 to 20 ms and 2.0 to 18.6 ms. Room temperature data were collected with the sample contained in a cylindrical 7 mm diameter thin walled vanadium can, located in front of the back-scattering detectors, while for elevated temperatures, samples were sealed in a silica tube placed inside an 11 mm diameter thin walled vanadium can. Data were collected at 50 °C intervals from 300 °C to 700 °C. Long data collections of 1000 μA h were made at room temperature, 500 °C and 700 °C to allow for total scattering analysis. Short data collections of 30 μA h were carried out at all other temperatures. For total scattering data correction, diffraction data were collected on an empty silica tube inside a thin walled vanadium can for *ca.* 700 μA h, under identical conditions to the sample at 500 °C and 700 °C.

Average structure refinement was carried out by conventional Rietveld analysis, with the GSAS suite of programs.^25^ A cubic model in space group *Fm*3̄*m* was used for all refinements. Bi, Pb and Y were located on the ideal 4a site (0, 0, 0), with oxide-ions distributed over three sites: 8c at (0.25, 0.25, 0.25); 32f at approximately (0.3, 0.3, 0.3) and 48i at around (0.5, 0.2, 0.2).^26^ A total oxide-ion occupancy constraint was applied and thermal parameters for the oxide-ions tied to a single value. The crystal and refinement parameters for the room temperature, 500 °C and 700 °C analyses are summarized in Table 1.

**Table 1:** Crystal and refinement parameters for δ-Bi_5_PbY_2_O_11.5_ at selected temperatures^b^

Temperature	20 °C	500 °C	700 °C
Chemical formula	Bi_5_PbY_2_O_11.5_	Bi_5_PbY_2_O_11.5_	Bi_5_PbY_2_O_11.5_
Formula weight	1613.905 g mol^−1^	1613.905 g mol^−1^	1613.905 g mol^−1^
Crystal system	Cubic	Cubic	Cubic
Space group	*Fm*3̄*m*	*Fm*3̄*m*	*Fm*3̄*m*
Lattice parameter	*a* = 5.48536(1) Å	*a* = 5.51978(1) Å	*a* = 5.54246(1) Å
Volume	165.050(1) Å^3^	168.176(1) Å^3^	170.258(1) Å^3^
*Z*	C2	0.5	0.5
Density (calc)	8.119 mg m^−3^	7.968 mg m^−3^	7.870 mg m^−3^
Sample description	Yellow powder	Yellow powder	Yellow powder
*R*-factors^a^	(a) Neut. backscatter.		
	*R*_wp_ = 0.0169	*R*_wp_ = 0.0055	*R*_wp_ = 0.0053
	*R*_p_ = 0.0276	*R*_p_ = 0.0097	*R*_p_ = 0.0101
	*R*_ex_ = 0.0053	*R*_ex_ = 0.0052	*R*_ex_ = 0.0058
	*R*_F_^2^ = 0.0556	*R*_F_^2^ = 0.0584	*R*_F_^2^ = 0.0629
	(b) Neut. low angle		
	*R*_wp_ = 0.0265	*R*_wp_ = 0.0106	*R*_wp_ = 0.0110
	*R*_p_ = 0.0225	*R*_p_ = 0.0100	*R*_p_ = 0.0103
	*R*_ex_ = 0.0149	*R*_ex_ = 0.0128	*R*_ex_ = 0.0139
	*R*_F_^2^ = 0.0555	*R*_F_^2^ = 0.0337	*R*_F_^2^ = 0.0288
No. of variables	87	87	87
No. of profile points used	3347 (neut. backscatter.)	3347 (neut. backscatter.)	3347 (neut. backscatter.)
	3467 (neut. low angle)	3467 (neut. low angle)	3257 (neut. low angle)

a For definition of *R*-factors see Larson and Von Dreele.^25^

b Estimated standard deviations are given in parentheses.

### 2.3 Total neutron scattering analysis

**Fig. 1:**
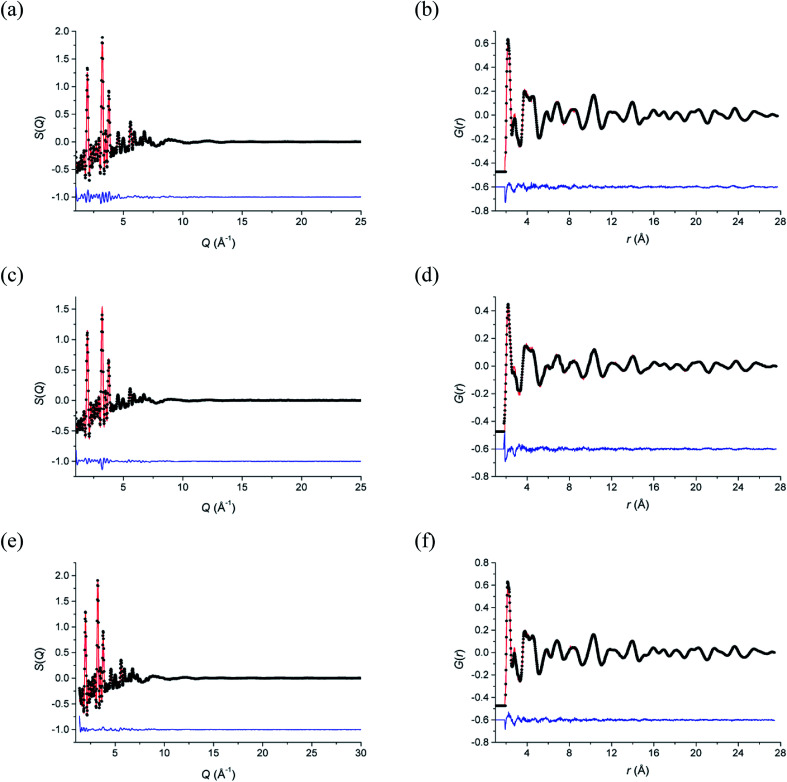
Fitted (a, c and e) total scattering *S*(*Q*) and (b, d and f) total radial distribution *G*(*r*) functions for δ-Bi_5_PbY_2_O_11.5_ at (a and b) 700 °C, (c and d) 500 °C and (e and f) 20 °C. Observed (points), calculated (line) and difference (lower) distributions are shown.

Total neutron scattering analysis using reverse Monte Carlo (RMC) modelling was used to examine the short-range ion pair correlations at room temperature, 500 °C and 700 °C. The program Gudrun^27^ was used to correct for background scattering and beam attenuation. The resulting normalized total scattering structure factors, *S*(*Q*), were then used to obtain the corresponding total radial distribution function, *G*(*r*), *via* Fourier transformation. The analysis of the total scattering data (Bragg peaks plus diffuse scattering components) was carried out using the RMCProfile software.^28^ Initial configurations of 10 × 10 × 10 unit cells were used in calculations, based on the ideal fluorite structure, with cations and anions randomly distributed over sites in the supercell, corresponding to the regular 4a and 8c crystallographic sites, respectively, in the cubic *Fm*3̄*m* subcell. At each of the studied temperatures, ten parallel configurations were analysed, each comprising of a different initial random configuration of atoms. Fitting was carried out against *S*(*Q*) and *G*(*r*), as well as the Bragg profile data to provide a constraint for the long-range crystallinity. *S*(*Q*) data were broadened by convolution with a box function to reflect the finite size of the simulation box, as previously described.^29^ Bond valence summation (BVS) constraints^30^ and an O–O potential constraint were applied during calculations. Cation swapping, *i.e.* the swapping of one cation with a randomly selected cation of another type was applied throughout the calculations. Further details on the background to the total scattering method are discussed by Keen.^31^ The fitted *S*(*Q*) and *G*(*r*) data are given in Fig. 1 and confirm that the RMC models accurately describe the scattering data in both reciprocal space and real space, respectively.

### 2.4 DFT simulations

Density functional theory calculations were performed using the Vienna Ab initio Simulation Package (VASP) within a plane wave basis set^32,33^ and with exchange–correlation treated within the generalized gradient approximation (GGA) of Perdew–Burke–Ernzerhof (PBE).^34^ We have found that the GGA functional offers the best compromise between calculation efficiency and accuracy for these types of systems.^35^ The electron–ion interactions were described by the full potential projector augmented wave (PAW) method,^36^ with 5, 6, 4 and 11 valence electrons for Bi, O, Pb and Y (including 4s and 4p states as valence for the latter), respectively. Forces in the system were calculated using the Hellmann–Feynman theorem^37,38^ and the kinetic energy was controlled by means of the Nose–Hoover thermostat.^39,40^ The plane wave cut-off energy was 450 eV for all calculations (value based on initial simulation optimization). The sampling of the Brillouin zone was performed using the Monkhorst–Pack scheme.^41^ All calculations were performed under periodic boundary conditions.

Structure relaxation was carried out for different vacancy ordering configurations using a random vacancy/cation configuration in a 2 × 2 × 2 supercell (78 atoms), with a 2 × 2 × 2 k mesh. The MD simulations used the relaxed 2 × 2 × 2 supercell at the gamma point. The global break point for the electronic loop was set to 10^−5^ eV per cell.

The lattice parameters used were those obtained from Rietveld refinement of the neutron data at the respective temperatures. The atomic starting positions were based on those in the ideal fluorite structure. In the ideal fluorite structure cations are located at the corners and faces of the unit cell. Therefore, to ensure no implied cation ordering and the correct stoichiometry in the supercell, the coordinates in the initial model were shifted by (¼, ¼, ¼) with respect to the ideal fluorite structure and the lead and yttrium atoms placed pseudo-randomly within this model. Structure relaxation was carried out at 0 K, while MD simulations were carried out at 25, 500 and 700 °C. Five parallel calculations were performed at each temperature. Calculations were carried out over a total of 60 ps in steps of 10 fs. Mean square displacements (MSDs) were calculated using eqn (1):1
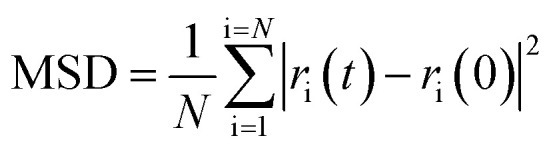
where *N* is the number of oxide-ions in the simulated cell and *r*_i_(*t*) and *r*_i_(0) are the position of ion i at time *t* and initial time, respectively. The diffusion coefficient was determined from the slope of the MSD curve by least squares fitting. To avoid the influence of thermal equilibration on the derived results, the first 10 ps of the MD simulations were ignored. The time step used was based on the results of our previous studies on related systems,^35,42,43^ which showed 10 fs to give equivalent accuracy in calculations compared to shorter time steps, when used with a suitable cut-off energy.

Pair distributions, contact distances, coordination numbers and ionic density profiles were calculated from the MD simulations by summation over all time steps and all five different initial random distributions of yttrium/lead cations and oxygen vacancies at each studied temperature. To clarify oxide-ion jump trajectories, high frequency oscillations of the oxide-ions were excluded from calculations using a low pass Chebyshev filter, with a cut-off frequency of 5 × 10^12^ Hz, as previously described.^44^ This approach excludes short unsuccessful jumps from the analysis and highlights the diffusion pathways.

## 3. Results and discussion

### 3.1 Average structure analysis

**Fig. 2:**
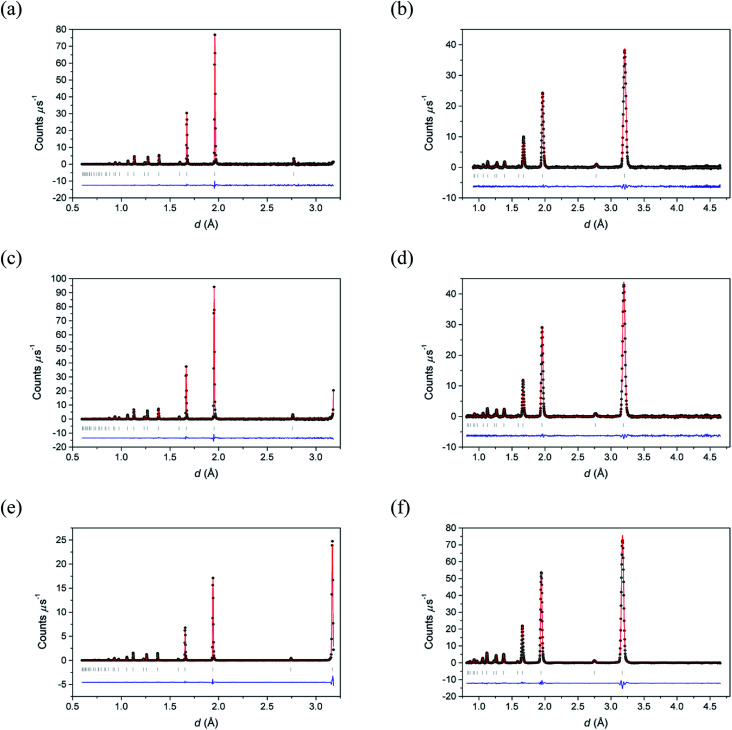
Fitted (a, c and e) neutron backscattering and (b, d and f) neutron low angle diffraction patterns for δ-Bi_5_PbY_2_O_11.5_ at (a and b) 700 °C, (c and d) 500 °C and (e and f) 20 °C. Observed (points), calculated (line) and difference (lower) profiles are shown. Reflection positions are indicated by markers.

Fig. 2 shows the neutron diffraction profiles for δ-Bi_5_PbY_2_O_11.5_ fitted by conventional Rietveld analysis at room temperature, 500 °C and 700 °C, with the corresponding refined structural parameters and significant contact distances given in Table 2. The data show that the fluorite structure is maintained throughout the temperature range studied, with no evidence of superlattice ordering, a phenomenon that occurs widely in the substituted bismuth oxides (see for example ref. 45–47). Instead, the diffraction patterns show broad background features, characteristic of short-range order, that can be probed through neutron total scattering analysis.

**Table 2:** Final refined structural parameters and significant contact distances for δ-Bi_5_PbY_2_O_11.5_ at selected temperatures^a^

Temperature	20 °C	500 °C	700 °C
Bi/Y/Pb site	4a	4a	4a
Bi/Y/Pb (*x*, *y*, *z*)	0.0	0.0	0.0
Bi/Y/Pb occ.	0.625/0.25/0.125	0.625/0.25/0.125	0.625/0.25/0.125
Bi/Y/Pb *U*_iso_ (Å^2^)	0.0459(1)	0.0573(2)	0.0667(2)
O(1) site	8c	8c	8c
O(1) (*x*, *y*, *z*)	0.25	0.25	0.25
O(1) occ.	0.412(6)	0.362(9)	0.358(11)
O(2) site	32f	32f	32f
O(2) (*x*, *y*, *z*)	0.3087(9)	0.3083(11)	0.3098(14)
O(2) occ.	0.063(2)	0.072(2)	0.070(3)
O(3) site	48i	48i	48i
O(3) (*x*)	0.5	0.5	0.5
O(3) (*y*, *z*)	0.1955(16)	0.1847(15)	0.1770(13)
O(3) occ.	0.009(1)	0.012(1)	0.014(1)
O(1)/O(2)/O(3) *U*_iso_ (Å^2^)	0.0621(6)	0.0682(8)	0.0749(10)
Bi/Y/Pb–O(1) (Å)	2.37523(1)	2.39013(1)	2.39996(1)
Bi/Y/Pb–O(2) (Å)	2.2516(8)	2.2661(11)	2.2740(14)
Bi/Y/Pb–O(3) (Å)	1.985(2)	2.017(3)	2.041(3)

a Estimated standard deviations are given in parentheses.

**Fig. 3:**
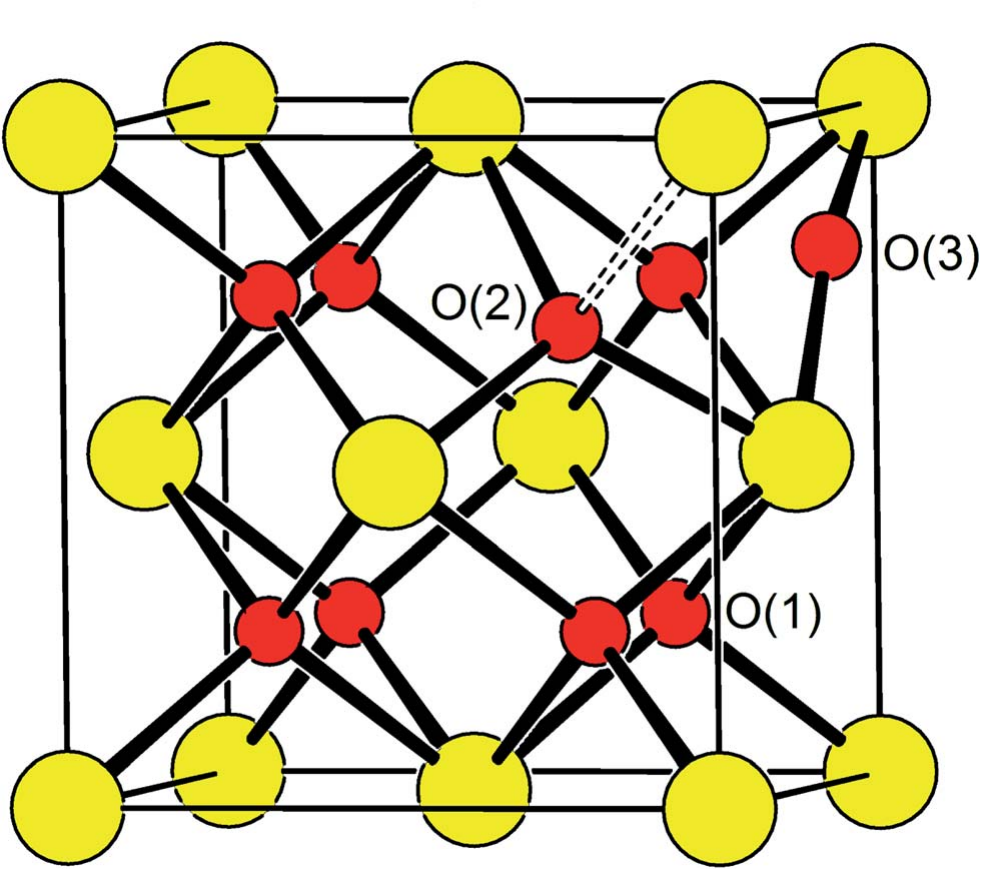
Idealized structure of δ-Bi_5_PbY_2_O_11.5_, showing relative positions of O(1), O(2) and O(3) sites. Disorder on oxide-ion sites has been omitted for clarity. Cation and oxide-ion positions are indicated by large (yellow) and small (red) spheres, respectively.

Oxide-ions are distributed over three crystallographically distinct sites, O(1), O(2) and O(3) corresponding to the 8c, 32f and 48i sites, respectively (Fig. 3). O(1) and O(2) sites are both contained within the tetrahedral cavity of the ccp lattice and are too close to each other to be simultaneously occupied. The O(1) site represents the centre of the tetrahedral site, while the O(2) site is shifted towards three of the four surrounding cations and so effectively reduces the average cation coordination number. The O(3) site is not seen to be occupied in the parent δ-Bi_2_O_3_ and therefore it is likely that ions on this site are exclusively associated with the dopant cations. Oxide-ions on the O(3) site are coordinated to two metal atoms only and lie outside the tetrahedral cavity of the ccp lattice. Ions on this site may therefore be considered as Frenkel interstitials, which effectively increase the vacancy concentration in the tetrahedral cavities. Since oxide-ions on the O(1) site are coordinated to four cations, those on the O(2) site are coordinated to three cations and those on the O(3) site coordinated to two cations, the weighted average cation coordination number 
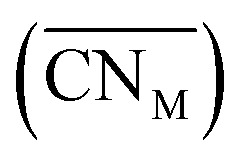
 can be calculated as:2
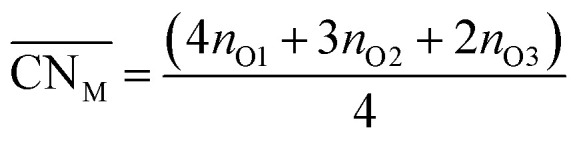
where *n*_O*i*_ is the number of O*i* (*i* = 1, 2, 3) atoms per cell. This gives 
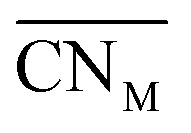
values of 5.02, 4.91 and 4.88 at room temperature, 500 °C and 700 °C, respectively.

**Fig. 4:**
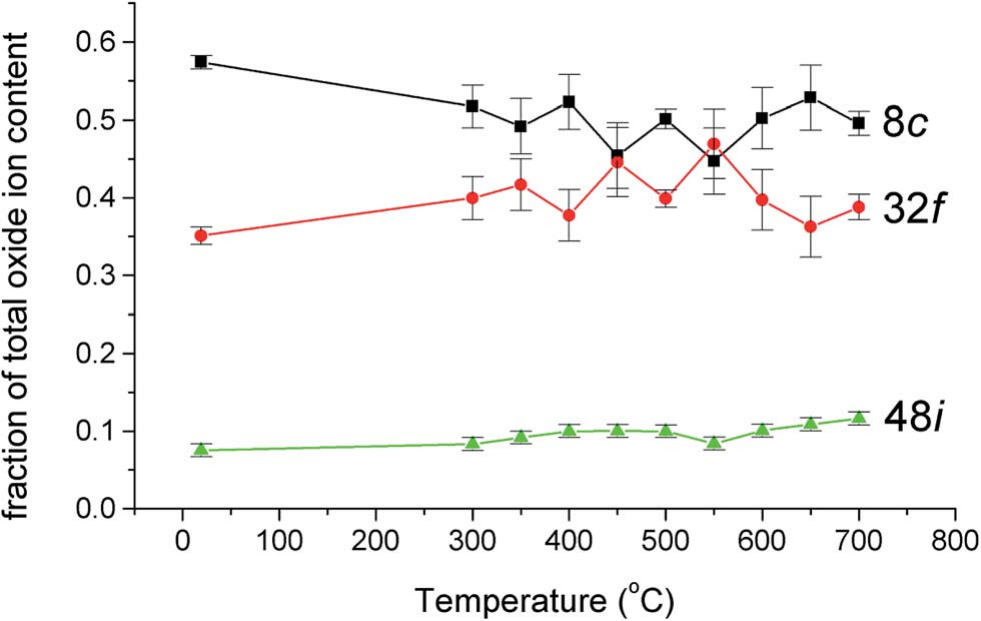
Thermal variation of fraction of total oxide-ion content for the three oxide sites identified in δ-Bi_5_PbY_2_O_11.5_.

The thermal variation of the fraction of the total oxide-ion content for the three oxide-ion sites in this system is shown in Fig. 4. From room temperature to *ca.* 500 °C the 32f site occupancy increases at the expense of that of the 8c site, but above this temperature there is little significant change. It is also evident that the number of oxide-ions on the 48i site shows a general increase with increasing temperature.

**Fig. 5:**
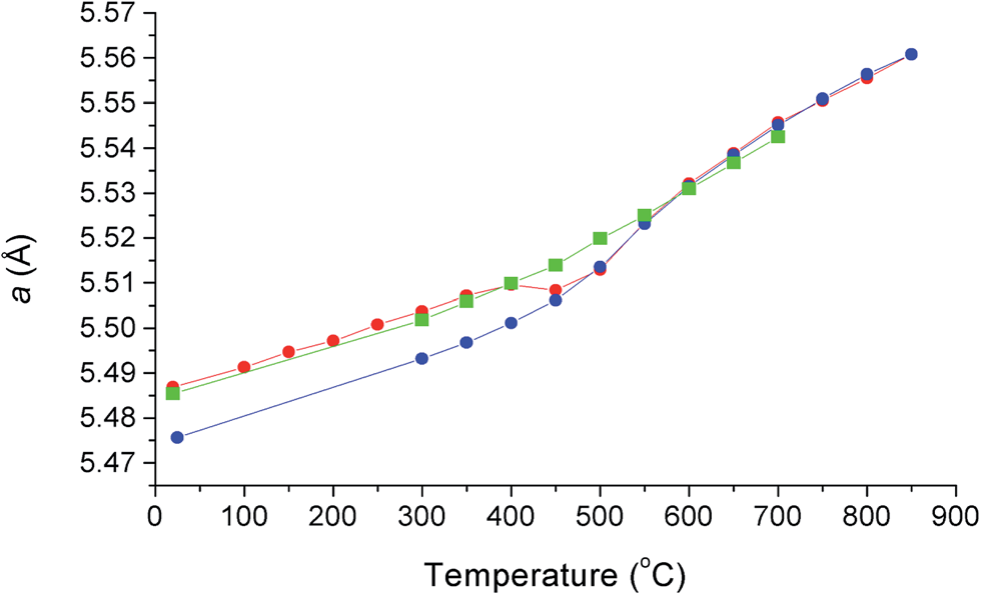
Thermal variation of cubic lattice parameter, *a*, for δ-Bi_5_PbY_2_O_11.5_, showing data collected using X-ray diffraction on heating in air (red circles), on cooling in air (blue circles) and using neutron diffraction on heating under vacuum (green squares). X-ray data taken from Borowska-Centkowska *et al.*^12^

The thermal variation of cubic lattice parameter on heating under vacuum is shown in Fig. 5 and is compared with previously obtained results from X-ray diffraction on heating and cooling in air.^12^ There are significant differences in the results from the two experiments, which can be attributed to small differences in oxygen stoichiometry, resulting from the different experimental conditions, underlining the sensitivity of this system to changes in oxygen partial pressure. The results from the present study, collected under vacuum, feature two linear regions above 150 °C, with a transition at around 450 °C. In the X-ray study in air, a significant difference was seen in the heating and cooling curves below 450 °C, associated with small changes in oxygen stoichiometry. The room temperature value of the lattice parameter in the present study is close to the value seen in the X-ray experiment on heating. Above 550 °C, the lattice parameter values from X-ray and neutron experiments are similar, with those from the present study slightly smaller due to increased reduction as a result of the reduced oxygen partial pressure in the evacuated sealed silica tube. The change in slope in the neutron data at around 450 °C, is a feature common in δ-phase bismuth oxide based systems and has been previously correlated with changes in oxide-ion/vacancy distributions.^29,48–50^

### 3.2 Local structure analysis

**Fig. 6:**
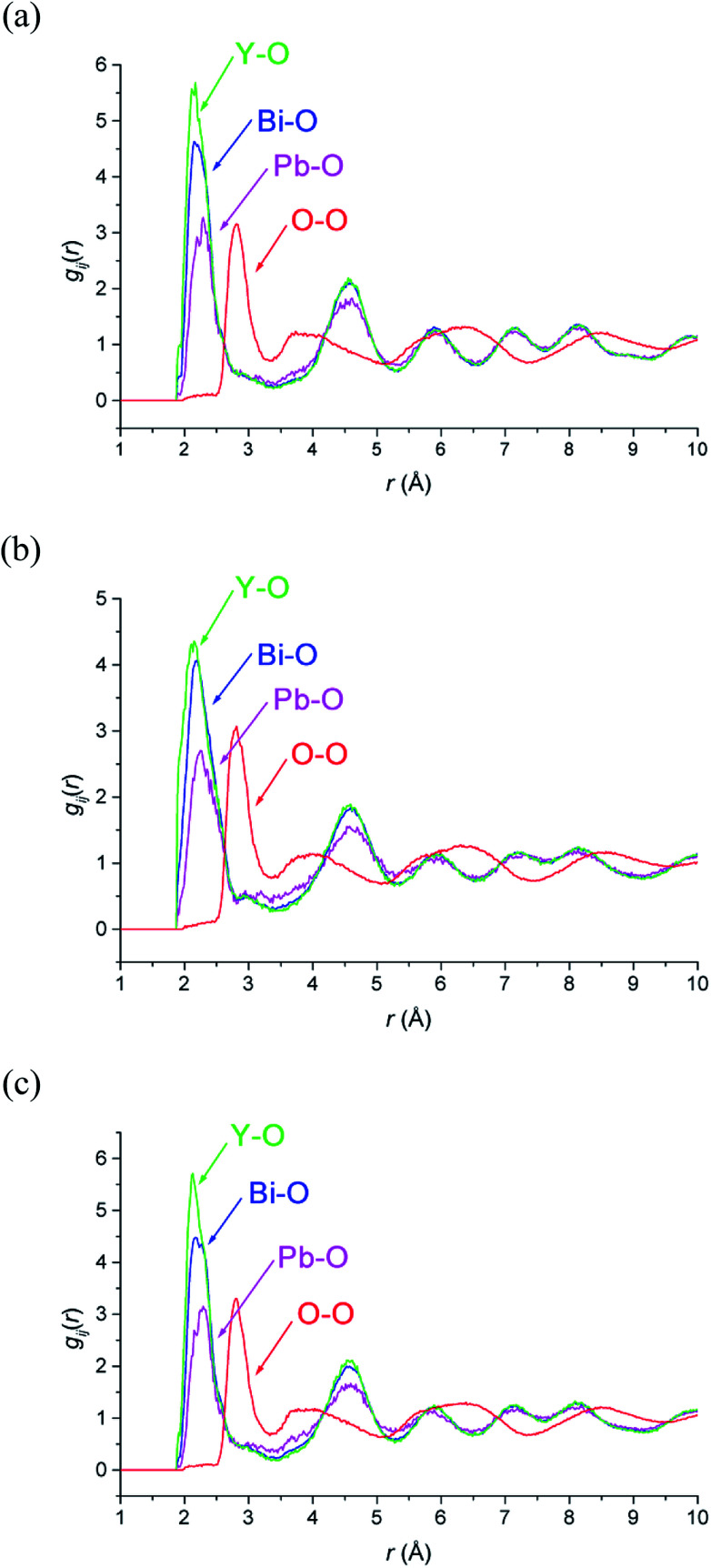
Selected ion pair correlation functions, *g*_ij_(*r*), derived from RMC models for δ-Bi_5_PbY_2_O_11.5_, at (a) 700 °C, (b) 500 °C and (c) 20 °C showing *g*_BiO_(*r*) (blue), *g*_PbO_(*r*) (magenta), *g*_YO_(*r*) (green), and *g*_OO_(*r*) (red) distributions.

M–O and O–O pair correlation functions, *g*_ij_(*r*), derived from the RMC analyses at 20, 500 and 700 °C are shown in Fig. 6. The final bond valence sums (BVS) along with the M–O coordination numbers and modal and mean contact distances are given in Table 3. The BVS values are all very close to those expected. There is little variation in distances and coordination numbers between temperatures from the RMC analyses. Two types of coordination number can be defined depending on the type of interactions being considered. The site coordination number is based on integration of the *g*_MO_(*r*) curve up to 3.30 Å, corresponding approximately to the point at which the curve flattens out, and takes into account all M–O contacts up to this distance, including bonding and non-bonding interactions. The resulting site coordination number gives the number of oxide-ions surrounding the cation site and hence for randomly distributed oxide-ions should agree with the theoretical value of 5.75, based on the stoichiometry of the title compound. This is only seen in the case of Bi^3+^ cations, with site coordination numbers of around 4.5 and 6 for Pb^2+^ and Y^3+^ cations, respectively. A local coordination number may also be calculated through integration up to the first minimum in the *g*_MO_(*r*) plots (Fig. 6) and gives a good approximation to the number of oxide-ions in the immediate coordination sphere of the cations. This includes both strong covalent interactions, as well as weaker interactions that may be considered to be non-bonding. The results in Table 3 show local coordination numbers of around 5 for Bi^3+^, 5.5 for Y^3+^ and 3.5 for Pb^2+^. The weighted average M–O local coordination numbers of 4.98(4), 4.85(3) and 4.89(2) at 20, 500 and 700 °C, respectively, are in good agreement with the average values obtained from the Rietveld analysis. To allow direct comparison with the crystallographic study, both mean and modal contact distances are presented in Table 3. The modal contact distances are all significantly lower than the mean values. This can be explained by considering that to allow direct comparison between the results obtained at the three studied temperatures, M–O distances were all calculated up to 2.83 Å and hence the mean values include both strong covalent bonds, as well as weaker interactions. Only in the case of the Bi–O distance is the mean value close to the sum of the ionic radii (2.41 Å, 2.57 Å and 2.28 Å for Bi–O, Pb–O and Y–O, respectively, based on six-fold coordination for cations^51^), with those for Pb–O slightly lower and those for Y–O slightly higher than the sum of the respective ionic radii. The weighted average M–O distances from the Rietveld analyses were 2.302, 2.303 and 2.309 Å, for 20, 500 and 700 °C experiments, respectively. These values are all slightly lower than the weighted averages of the mean values from the RMC calculations given in Table 3.

**Table 3:** Bond valence sums (BVS), M–O coordination numbers (CN) and modal and mean M–O contact distances (Å) from RMC analysis of δ-Bi_5_PbY_2_O_11.5_ at 20, 500 and 700 °C^a^

		20 °C	500 °C	700 °C
BVS	Bi^3+^	3.001(4)	2.981(3)	2.975(3)
	Pb^2+^	2.008(8)	1.994(8)	1.983(5)
	Y^3+^	2.983(7)	2.965(6)	2.958(3)
	O^2−^	1.998(1)	1.985(2)	1.980(2)
Site CN	Bi–O	5.76(6)	5.77(3)	5.62(2)
	Pb–O	4.48(10)	4.55(8)	4.32(10)
	Y–O	6.30(15)	6.20(8)	6.24(3)
Local CN	Bi–O	5.01(5)	4.92(3)	4.91(2)
	Pb–O	3.54(3)	3.49(8)	3.51(6)
	Y–O	5.61(12)	5.37(8)	5.54(4)
Distances	Bi–O mode	2.19(4)	2.179(6)	2.17(2)
	Bi–O mean	2.402(5)	2.422(3)	2.394(2)
	Pb–O mode	2.29(3)	2.21(3)	2.26(4)
	Pb–O mean	2.480(10)	2.500(7)	2.462(10)
	Y–O mode	2.12(1)	2.15(2)	2.13(1)
	Y–O mean	2.365(11)	2.385(5)	2.365(2)
	Av. M–O mode	2.19(2)	2.176(8)	2.17(2)
	Av. M–O mean	2.402(1)	2.422(1)	2.395(1)

a Values are averages of 10 parallel calculations and standard deviations are given in parentheses. M–O distances and local coordination numbers were calculated up to a maximum of 2.83 Å. Site coordination numbers were derived by integration of *g*_MO_(*r*) to a maximum of 3.30 Å.

**Fig. 7:**
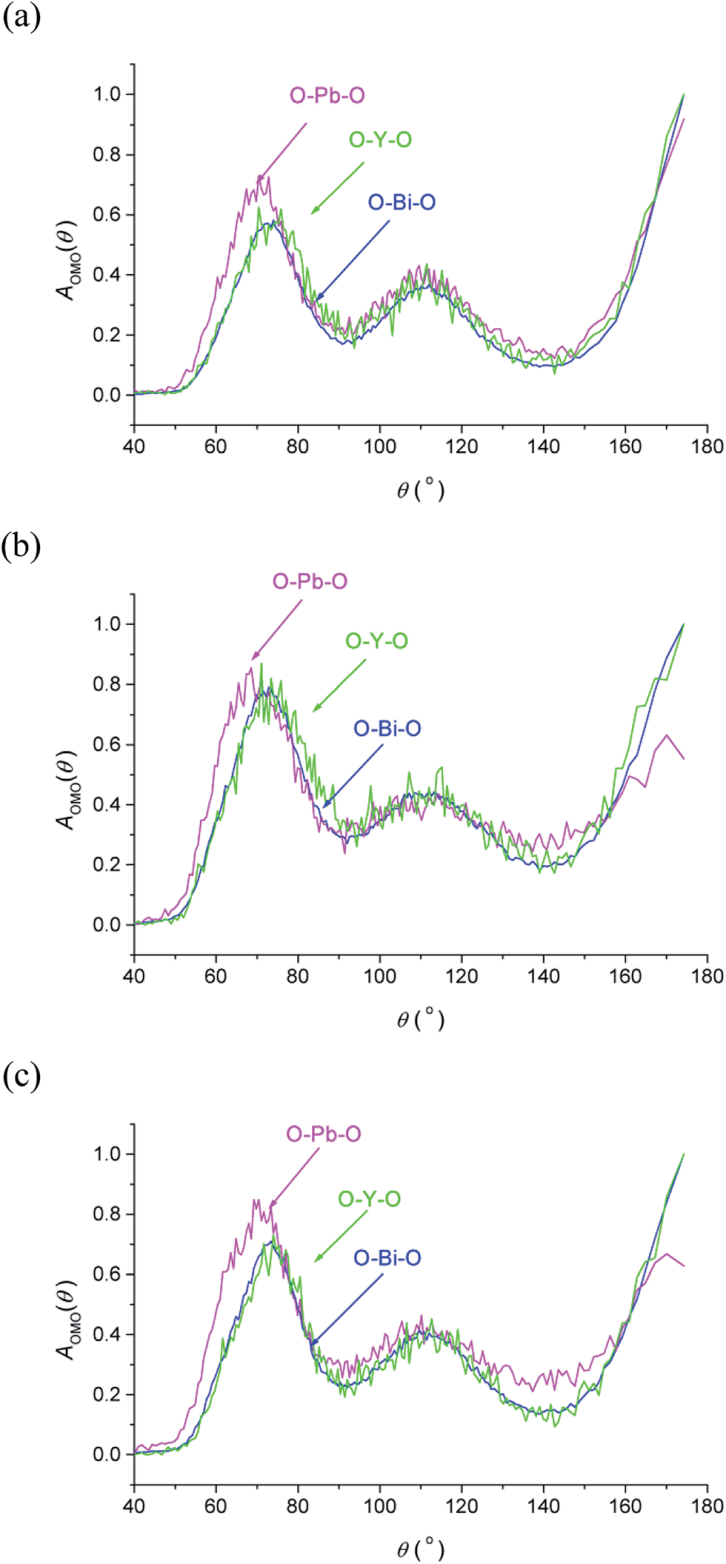
O–M–O angular distribution functions, *A*_OMO_(*θ*), derived from RMC models for δ-Bi_5_PbY_2_O_11.5_, at (a) 700 °C, (b) 500 °C and (c) 20 °C, showing *A*_OBiO_(*θ*) (blue), *A*_OPbO_(*θ*) (magenta) and *A*_OYO_(*θ*) (green) distributions.

The O–M–O angular distribution functions *A*_OMO_(*θ*) derived from the RMC configurations at the three studied temperatures are shown in Fig. 7. The plots show three maxima corresponding to the angles between 〈100〉, 〈110〉 and 〈111〉 aligned oxide-ion pairs in the fluorite structure. The O–Bi–O and O–Y–O distributions are similar to each other and compare well with those in δ-Bi_3_YO_6_.^29^ Interestingly, the O–Pb–O distribution is significantly different, particularly at lower angles, where at room temperature a peak maximum is seen at around 70°, which is approximately 6° lower than seen for the other two distributions. This difference suggests a greater local distortion away from the fluorite structure around Pb^2+^ than around the other cations. This distortion is likely to be correlated with the extent of stereochemical activity of the Pb^2+^ 6s^2^ lone pair, compared to that of Bi^3+^.

Unlike the average structure determined using Rietveld analysis, the final RMC configuration can readily be examined for vacancy concentration and vacancy ordering as previously demonstrated.^48^ Vacancies were assumed to sit at the centre of the tetrahedral site in the starting configuration. Sites that contained no oxide-ions up to a distance of 1 Å were deemed to be vacant. This value was based on the O(1)⋯O(3) and O(1)⋯O(2) contact distances in the Rietveld analyses to specifically take into account O(2) ions which lie within the tetrahedral cavity, but exclude O(3) ions which sit in an interstitial site. Once identified, the vacancy pair correlations were examined. Table 4 summarizes the defect distribution parameters obtained from the RMC calculations. At each temperature, the tetrahedral vacancy concentration is considerably higher than the theoretical value of 2.25 per fluorite cell and increases with increasing temperature. This is consistent with the results of the Rietveld analyses which show significant occupancy of the interstitial 48i site (O(3)), which increases with increasing temperature. These results are similar to those seen in δ-Bi_4_YbO_7.5_ (ref. 48) and δ-Bi_3_YbO_6_,^49^ with the vacancy concentrations seen in the present study slightly higher, reflecting the difference in oxygen stoichiometry.

**Table 4:** Defect concentration and distribution parameters derived from RMC analyses of δ-Bi_5_PbY_2_O_11.5_ at 20, 500 and 700 °C^a^

	20 °C	500 °C	700 °C	Theoretical
No. tet. vacancies per fluorite cell	3.02(2)	3.15(1)	3.40(2)	2.25
100 : 110 : 111	1.00 : 1.74 : 1.15	1.00 : 1.73 : 1.13	1.00 : 1.83 : 1.21	1.0 : 2.0 : 1.3
%vac(NN)_Bi_	47.9(9)	47.7(2)	47.5(5)	62.5
%vac(NN)_Pb_	26.1(4)	26.0(3)	25.8(4)	12.5
%vac(NN)_Y_	26.0(7)	26.4(3)	26.7(3)	25.0
%Bi(NNN)_Bi_	62.7(4)	62.6(2)	62.7(3)	62.5
%Bi(NNN)_Pb_	12.1(3)	12.0(2)	12.2(2)	12.5
%Bi(NNN)_Y_	25.2(2)	25.4(1)	25.1(2)	25.0
%Pb(NNN)_Bi_	61.5(1.5)	61.5(8)	61.8(5)	62.5
%Pb(NNN)_Pb_	17.2(1.2)	16.8(9)	17.3(8)	12.5
%Pb(NNN)_Y_	21.3(1.0)	21.7(8)	21.0(5)	25.0
%Y(NNN)_Bi_	62.7(5)	63.1(2)	62.2(6)	62.5
%Y(NNN)_Pb_	10.4(5)	10.5(4)	10.3(3)	12.5
%Y(NNN)_Y_	26.9(7)	26.3(5)	27.5(8)	25.0

a Values are averages of 10 parallel calculations and standard deviations are given in parentheses.

**Fig. 8:**
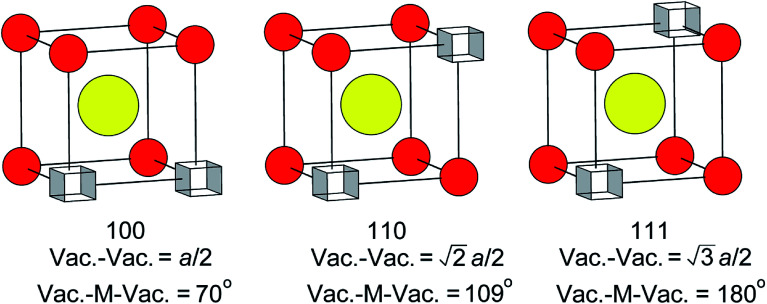
Possible vacancy pair alignments around cations in the ideal fluorite structure, with characteristic vacancy–vacancy distances and vacancy–metal–vacancy angles shown.

There has been much discussion in the literature regarding the ordering of oxide-ion vacancy pairs in bismuth oxide and related systems, particularly with respect to the energetics of oxide-ion motion.^52–60^ Three basic vacancy pair alignments can be defined with respect to the cubic coordination of the cation in the ideal fluorite structure, each characterized by a specific vacancy–vacancy distance and O–M–O angle (Fig. 8). Assuming a random distribution of vacancies, a ratio of 1 : 2 : 1.3 for 〈100〉 : 〈110〉 : 〈111〉 vacancy pairs is expected. The values given in Table 4 show significant departure from these ideal ratios, revealing a clear preference for 〈100〉 ordering compared to the random situation. There is little change in this ratio from ambient temperature to 500 °C. However, a more significant change is observed in the results at 700 °C, with the ratio closer to that in the random distribution. When considering the vacancy pair distributions one cannot ignore the high vacancy concentration in this system. Unlike dilute systems, in the present study, with around 3 tetrahedral vacancies per cell, it is impossible to accommodate 〈100〉 or 〈111〉 vacancy pair alignments exclusively.^48^ Therefore, one cannot consider a single vacancy pair alignment in isolation, but should take into account vacancy clusters involving three or more vacancies. These clusters can show various distributions of vacancy pairs and we have previously proposed a model that accommodates three vacancies per cell with a 〈100〉 : 〈110〉 : 〈111〉 vacancy pair ratio of 1 : 1.7 : 1.3,^48^ which is close to the ratios seen in the present study.

It is possible to reveal details of the local environment around vacancies through careful examination of the RMC configurations. Using a similar approach to that discussed above, the vacancy “coordination” environment can be probed to reveal evidence of a preferential distribution of vacancies between the cations. The nearest-neighbour cation distributions around oxide-ion vacancies are presented as percentages of the total number of nearest-neighbour cations in Table 4. A random distribution of cations would be expected to yield percentage distributions reflecting the stoichiometry, *i.e.* 62.5% Bi^3+^, 12.5% Pb^2+^ and 25% Y^3+^. The values in Table 4 for the percentage of cation nearest-neighbours around a vacancy, %vac(NN)_M_, show significant deviation from a purely random distribution. In particular, the high percentage of Pb^2+^ nearest-neighbours at all studied temperatures, strongly suggests preferential association of vacancies with this cation.

**Fig. 9:**
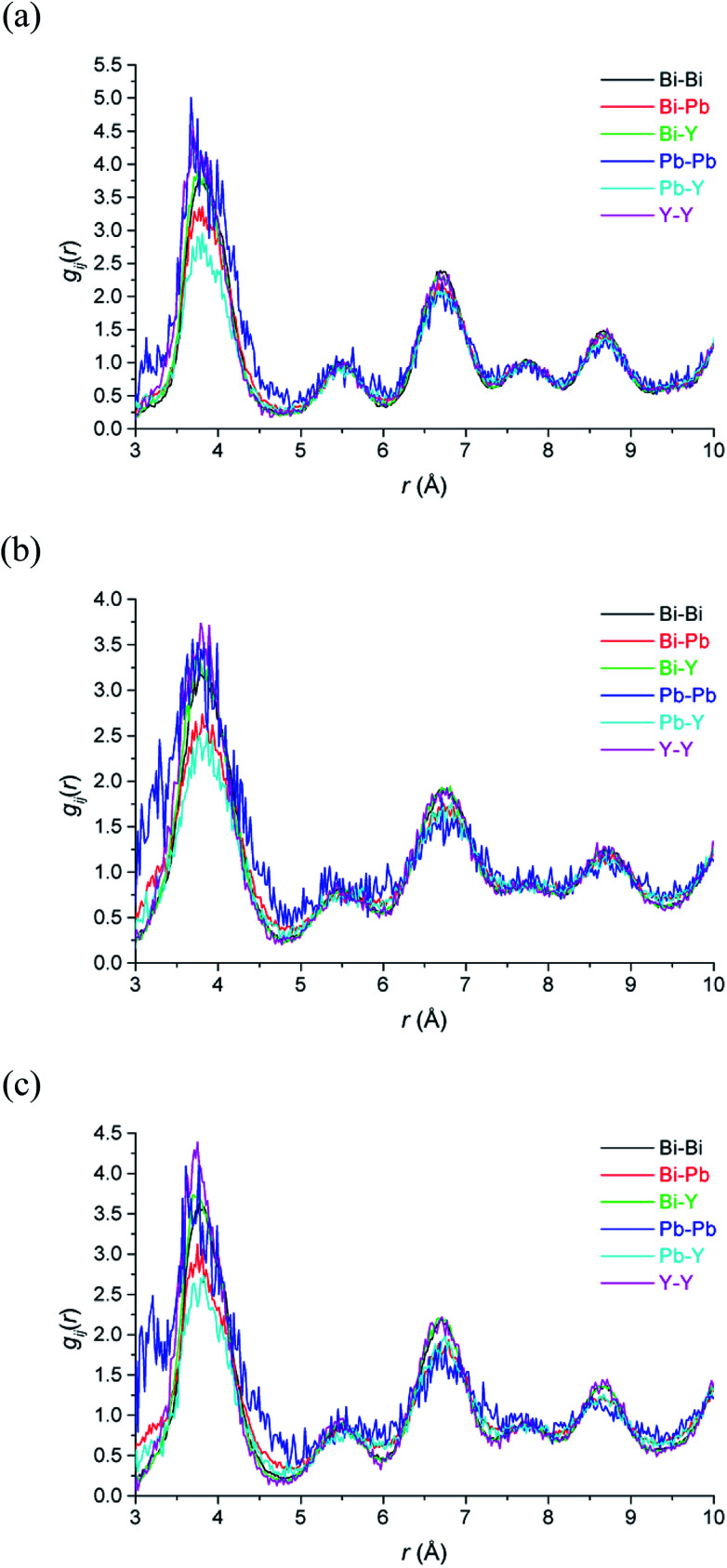
Cation–cation pair correlation functions, *g*_MM_(*r*), derived from RMC models for δ-Bi_5_PbY_2_O_11.5_, at (a) 700 °C, (b) 500 °C and (c) 20 °C showing *g*_BiBi_(*r*) (black), *g*_BiPb_ (*r*) (red), *g*_BiY_(*r*) (green), *g*_PbPb_(*r*) (blue), *g*_PbY_(*r*) (cyan) and *g*_YY_(*r*) (magenta) distributions.

The large RMC configurations also allow for the examination of the specific local environment of the different types of cations. This is particularly relevant in the case of systems where different types of cation share the same crystallographic site and hence are effectively indistinguishable in the average structure analysis. Fig. 9 shows the partial pair correlations for cation pairs. It is evident that cation pair correlations exhibit similar distributions to each other. Further detail emerges when the percentage of each type of cation next-nearest-neighbours around the different cation types, %M(NNN)_M_, at each of the studied temperatures, is calculated (Table 4). As discussed above, with respect to the cation distribution around vacancies, the theoretical percentages in a random distribution reflect the stoichiometry of the title compound. In the case of next-nearest-neighbours around Bi^3+^ cations, the distribution shows little deviation away from a random distribution. For Y^3+^ the next-nearest-neighbours include significantly fewer Pb^2+^ and slightly more Y^3+^ cations than expected. The biggest deviation from the random distribution is seen in the next-nearest-neighbours to Pb^2+^ cations. There are significantly more Pb^2+^ cations as next-nearest-neighbours to other lead cations than would be expected from a random distribution. The results suggest that some Pb^2+^ cations cluster in the cubic close packed lattice. There is also some evidence for clustering of Y^3+^ cations, which would be a prerequisite for transformation to the rhombohedral phase, known to occur in substituted bismuth oxides such as Bi_3_YO_6_ on annealing at temperatures around 650 °C.^17,18^

### 3.3 Simulations

**Fig. 10:**
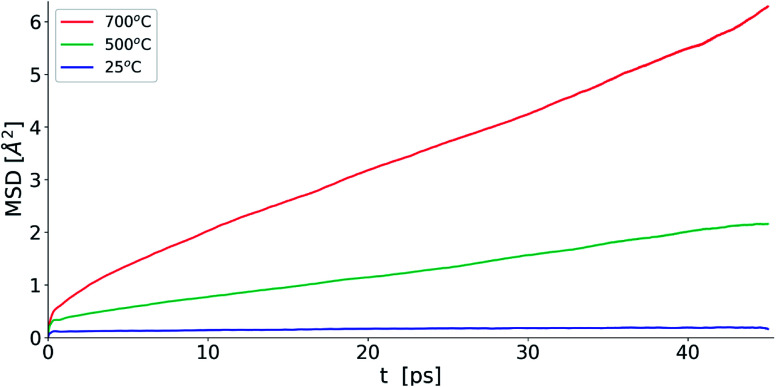
Oxide-ion MSD evolution for MD simulations for δ-Bi_5_PbY_2_O_11.5_ at selected temperatures.

**Fig. 11:**
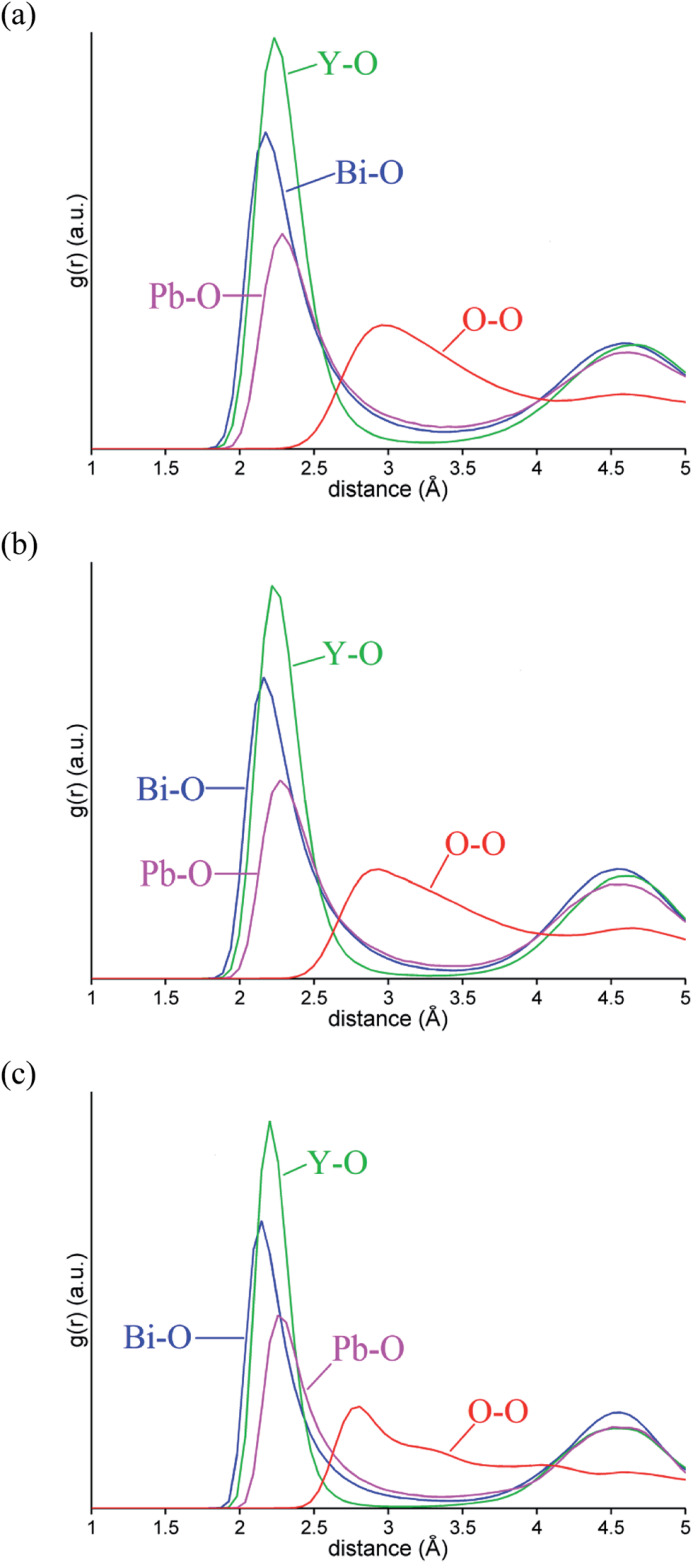
Selected ion pair correlation functions *g*_MO_(*r*) from MD simulations for δ-Bi_5_PbY_2_O_11.5_, at (a) 700 °C, (b) 500 °C and (c) 25 °C showing *g*_BiO_(*r*) (blue), *g*_PbO_(*r*) (magenta), *g*_YO_(*r*) (green), and *g*_OO_(*r*) (red) distributions.

Fig. 10 shows the evolution of MSD with simulation time at the three studied temperatures. As expected, the MSD at 25 °C is almost flat and increases with increasing temperature. From the slopes of the MSD plots, the diffusion coefficients at 500 and 700 °C were found to be 1.92 × 10^−7^ and 3.34 × 10^−7^ cm^2^ s^−1^, respectively and are lower than the value of 2.06 × 10^−6^ cm^2^ s^−1^ seen in δ-Bi_3_YO_6_ at 700 °C.^35^ Selected pair distribution functions derived from the simulations are shown in Fig. 11, with the derived coordination numbers and modal contact distances given in Table 5. The asymmetry of the *g*_PbO_(*r*) and *g*_BiO_(*r*) plots, reflects the asymmetric coordination environment of these cations and is also seen in the plots derived from the RMC study (Fig. 6). The Bi–O modal contacts are in good agreement between the two studies, while those for Pb–O are slightly higher in the MD study. The partial pair correlation plots for Y–O and O–O show some subtle differences. In the MD model the first Y–O peak is sharper and more symmetric than seen in the RMC calculations and the modal Y–O distance is larger than that obtained from the neutron study. This discrepancy may be in part due to the difficulty in discriminating the relative contributions of bismuth and yttrium in the neutron scattering study caused by the small neutron contrast between them (*b* = 7.75, 8.532 and 9.405 fm for Y, Bi and Pb, respectively). In contrast, the first O–O maximum in the *g*_OO_(*r*) plot in Fig. 6 is sharper than that seen in the MD simulations. This latter difference may to some extent be attributable to the O–O potential constraint used in the RMC calculations. The Bi–O and Y–O coordination numbers derived from the MD simulations of around 5 are similar to those from the RMC calculations, while the values for Pb–O of 4.4 to 4.5 are larger than seen in the RMC calculations.

**Table 5:** Cation coordination numbers to oxygen and modal contact distances (Å) for δ-Bi_5_PbY_2_O_11.5_ derived from MD simulations at the studied temperatures

	25 °C	500 °C	700 °C
Bi CN	4.942	4.952	4.985
Pb CN	4.504	4.372	4.425
Y CN	5.092	5.482	5.380
M CN	4.92	5.01	5.01
Bi–O	2.19	2.18	2.18
Pb–O	2.34	2.31	2.30
Y–O	2.25	2.25	2.26

**Fig. 12:**
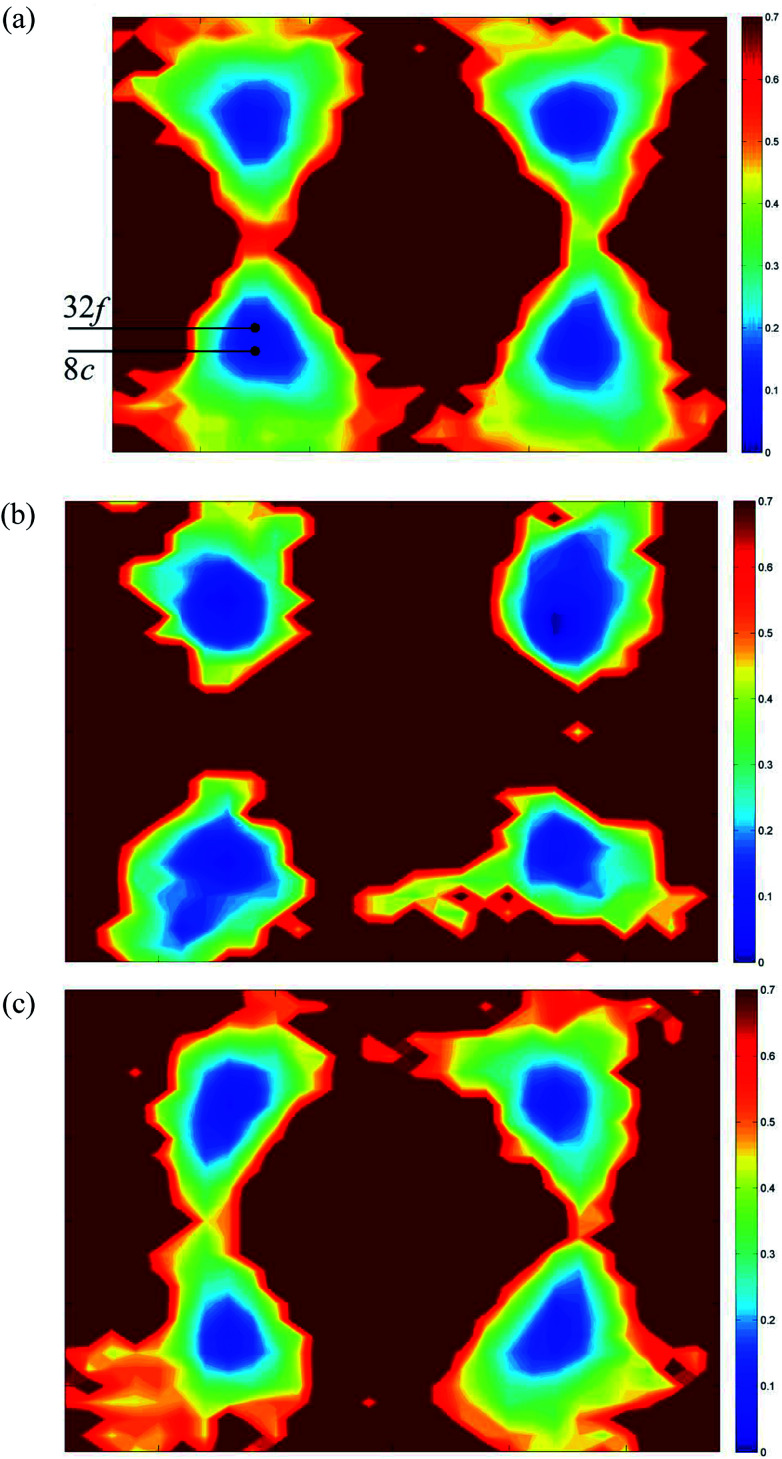
〈110〉 cross-sections of oxide-ion density around (a) Bi^3+^, (b) Pb^2+^ and (c) Y^3+^ cations in δ-Bi_5_PbY_2_O_11.5_.

**Fig. 13:**
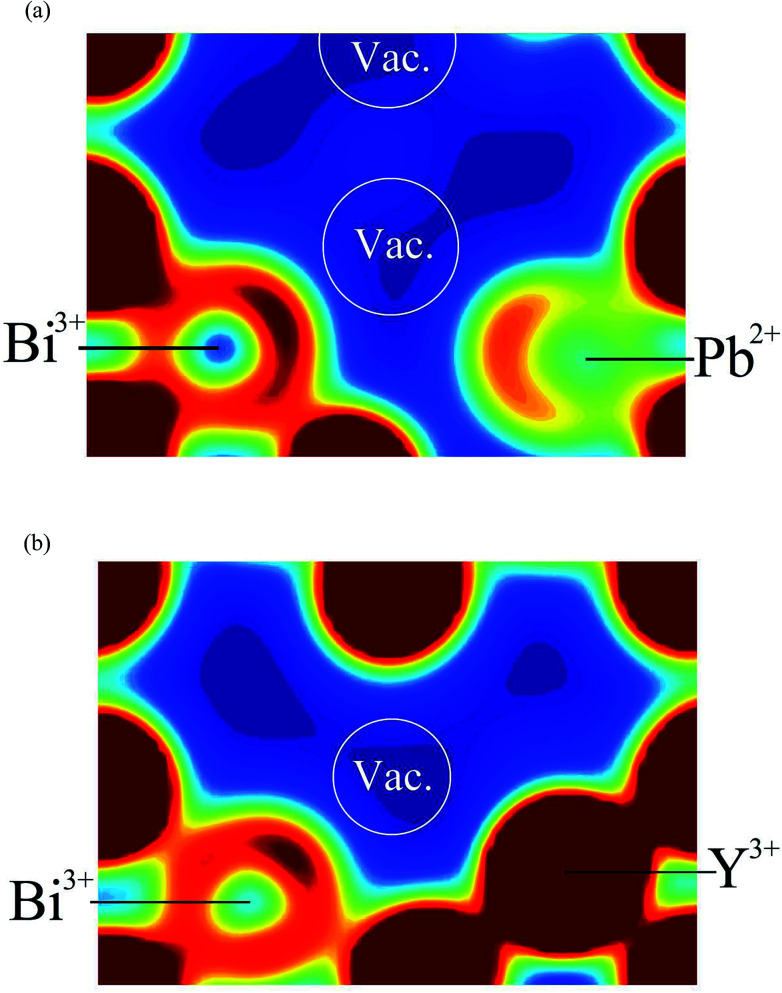
Electron density cross sections around single vacancies in δ-Bi_5_PbY_2_O_11.5_ showing states corresponding to the energy range −6 eV to 0 eV (Fermi energy set to 0 eV); (a) vacancy with Bi^3+^ and Pb^2+^ neighbours and (b) vacancy with Bi^3+^ and Y^3+^ neighbours.

Fig. 12 shows 〈110〉 cross-sections of ionic density around the different cations in δ-Bi_5_PbY_2_O_11.5_. It is evident that oxide-ion density is significantly less localized around Bi^3+^ than around Pb^2+^. The oxide-ion density around bismuth cations is spread out over the tetrahedral cavity, resulting in significant density at sites corresponding to both 8c and 32f in the crystallographic model, as indicated in Fig. 12a. The oxide-ion density neighbouring Pb^2+^ (Fig. 12b) is more localized around the 8c site at the centre of the tetrahedral cavity, with little discernable occupation of the 32f sites. Oxide-ion density around Y^3+^ (Fig. 12c) appears to be intermediate between that around Bi^3+^ and Pb^2+^. Fig. 13 shows charge density profiles around vacancies in δ-Bi_5_PbY_2_O_11.5_. In Fig. 13a, Bi^3+^ and Pb^2+^ ions are shown and for both ions the lone pairs are clearly evident and point to the vacancy. Fig. 13b shows the charge density around a vacancy, with Y^3+^ cation neighbours. The presence of the vacancy appears to have no discernable effect on the charge density around Y^3+^, which is close to spherical.

**Fig. 14:**
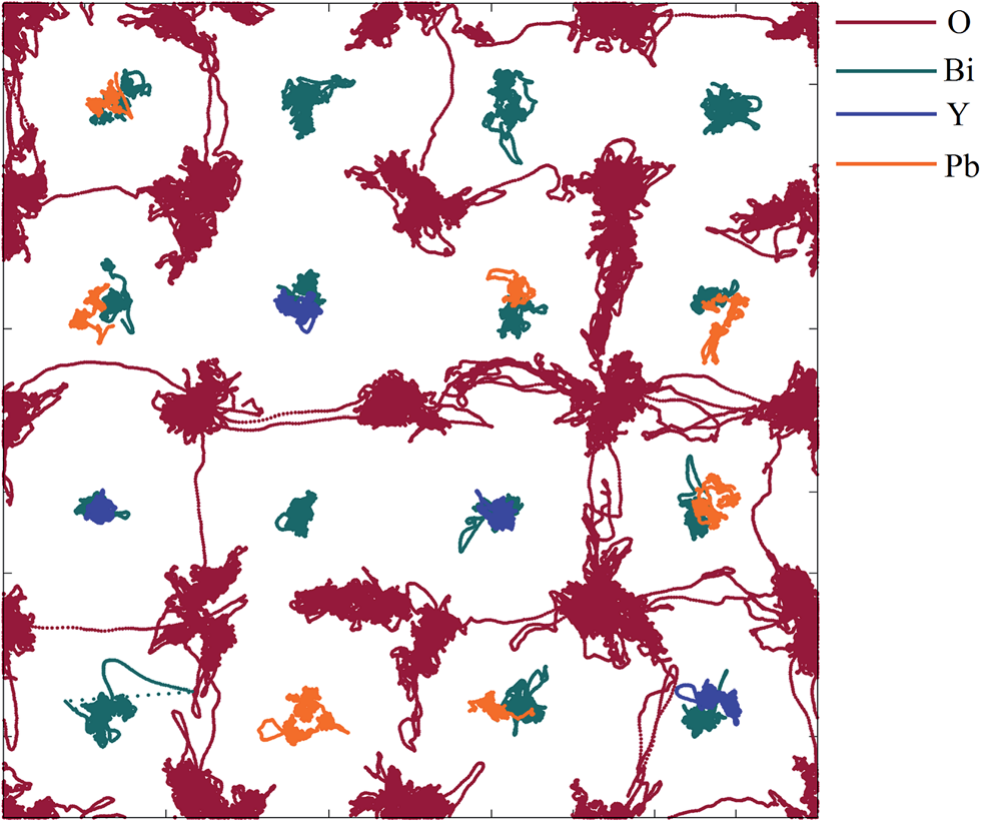
〈110〉 projection of ion trajectories in δ-Bi_5_PbY_2_O_11.5_ after processing with a low-pass Chebyshev filter. Individual ion types are denoted by different colours.

A projection of oxide-ion jump trajectories after application of a low-pass Chebyshev filter is shown in Fig. 14. The use of a low-pass Chebyshev filter effectively excludes thermal oscillations which do not contribute to long-range motion of ions. The trajectories are dominated by jumps in the 〈100〉 direction, which constitute 98% of all jumps observed, with 2% occurring in the 〈110〉 direction and none in the 〈111〉 direction. This is in good agreement with the results of similar calculations carried out on δ-Bi_3_YO_6_ (ref. 29) and δ-Bi_2_O_3_ (ref. 61 and 62) and is consistent with proposed models for ionic conductivity in δ-Bi_2_O_3_ based systems.^58–60,63,64^

### 3.4 Defect structure of Bi_5_PbY_2_O_11.5_

**Fig. 15:**
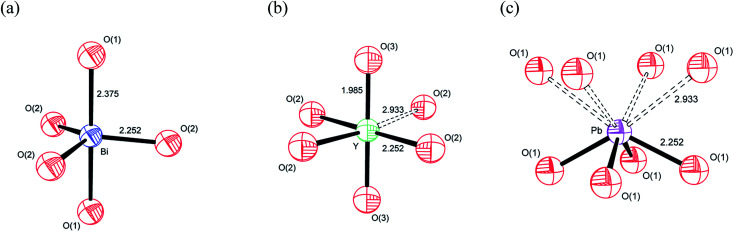
Proposed cation coordination geometries in δ-Bi_5_PbY_2_O_11.5_, showing (a) Bi^3+^, (b) Y^3+^ and (c) Pb^2+^ coordinations.

Based on the individual cation coordination numbers from the RMC and MD calculations and the average structure model from the Rietveld analysis, it is possible to propose models of the local coordination geometries adopted by the cations in δ-Bi_5_PbY_2_O_11.5_. Both MD and RMC studies suggest a coordination number of 5 for Bi^3+^ in the present system. Since it is known that occupation of the interstitial O(3) (48i) site does not occur in the parent δ-Bi_2_O_3_,^60–62,65–68^ the coordination sphere is likely to be made up of oxide-ions in the O(1) and O(2) sites only (Fig. 15a). Our previous studies have shown that in δ-Bi_3_YO_6_, there is occupation of the O(3) site.^29^ Therefore, it is likely, that in the present system, the Y^3+^ coordination sphere will involve oxide-ions on this site. Both MD and RMC studies show Y^3+^ to have a local coordination number of around 5.5. This could be achieved with Y^3+^ adopting both 5 and 6 coordinate geometries. It should be noted that the structural chemistry of Y^3+^ in oxide systems is more consistent with the more symmetrical six-fold coordination environment, as we have previously proposed.^29^ In the case of Y^3+^, which does not possess a stereochemically active lone pair, the site coordination number of around 6 may be more representative of the real situation, with Y^3+^ adopting a 5+1 six-fold coordination, as indicated in Fig. 15b by a dashed bond to a sixth oxide-ion. In the case of Pb^2+^ there is a significant difference between the local coordination number from the RMC calculations and that from the MD simulations, with the MD simulations yielding values close to those of the site coordination numbers from the RMC calculations, with values around 4. A model showing an asymmetric four-fold coordination for Pb^2+^ involving oxide-ions in the O(1) site is shown in Fig. 15c. While similar models can be obtained using O(2) or a combination of O(1) and O(2) sites, the ionic density plots from the MD study suggest that Pb^2+^ ions are coordinated to oxide-ions in the O(1) site.

## 4. Conclusions

As in other substituted δ-Bi_2_O_3_ based systems, δ-Bi_5_PbY_2_O_11.5_ shows oxide-ions distributed over three crystallographic sites, two of which are located within the tetrahedral cavity of the ccp lattice, with a third site, interstitial to the regular fluorite structure. Whilst there is little change in the gross structure, the oxide-ion distribution is found to vary significantly with temperature. A preference for 〈100〉 vacancy pair alignment is observed at room temperature and at 500 °C. At 700 °C, local oxide-ion vacancy ordering is still present, but is closer to a fully random distribution.

The approach to analysis of the RMC configurations developed in this study has uniquely afforded an opportunity for cation to oxide-ion vacancy associations to be probed. As a result a clear preference for vacancy association with lead cations has been identified. Additionally, this approach has facilitated the study of cation–cation interactions in a system that exhibits no long-range order of different cations. Pb^2+^ cations are found to cluster together, with some evidence of local ordering of Y^3+^ cations in this system. Oxide-ion motion is found to occur mainly in the 〈100〉 direction, consistent with the current understanding of oxide-ion transport in δ-Bi_2_O_3_ type systems.

## Conflicts of interest

There are no conflicts of interest to declare.

## Supplementary Material
